# Supplementary material for: Immunobiology and Cytokine Modulation of the Pediatric Brain Tumor Microenvironment: A Scoping Review
Source: Cancers (Basel). 2023 Jul 18;15(14):3655. doi: 10.3390/cancers15143655 (PMC10377457; doi:10.3390/cancers15143655)
Supplement: Supplementary file 1 [file cancers-15-03655-s001.zip › cancers-2459297-supplementary.pdf]

Supplementary Table S1: Search Terms and Articles reviewed

**Search Terms**	PUBMED: ("brain tumor" OR "brain tumour" OR "brain cancer") AND ("pediatric" OR "pediatrics" OR "paediatric" OR "paedatrics" OR "child" OR "children") AND ("immune" OR "immunobiology" OR "immunology" OR "immunotherapy")<br><br>SCOPUS: TITLE-ABS-KEY (("brain tumor" OR "brain tumour" OR "brain cancer") AND ("pediatric" OR "pediatrics" OR "paediatric" OR "paedatrics" OR "child" OR "children") AND ("immune" OR "immunobiology" OR "immunology" OR "immunity"))<br><br>EMBASE: ('brain tumor'/exp OR 'brain tumor' OR 'brain tumour'/exp OR 'brain tumour' OR 'brain cancer'/exp OR 'brain cancer') AND ('pediatric'/exp OR 'pediatric' OR 'pediatrics'/exp OR 'pediatrics' OR 'paediatric'/exp OR 'paediatric' OR 'paedatrics' OR 'child'/exp OR 'child' OR 'children'/exp OR 'children') AND ('immune'/exp OR 'immune' OR 'immunobiology'/exp OR 'immunobiology' OR 'immunology'/exp OR 'immunology' OR 'immunity'/exp OR 'immunity')

Article Title	Reference	Concept	Key Findings
Analysis of PD-L1 expression and T cell infiltration in different molecular subgroups of diffuse midline gliomas	Jha et al.	Pediatric brain development may elicit a different immune profile than in adults, T cell Exhastion	While CD3 infiltration in diffuse midline gliomas was similar in adults and children of all ages, CD8 expression was significantly higher in adults than in children. PD-L1 expression in DMGs was substantially correlated with the number of tumor-infiltrating lymphocytes, but it had no effect on survival.
NKG2D ligand expression in pediatric brain tumors	Haberthur et al.	Pediatric brain development may elicit a different immune profile than in adults	When compared to healthy neighboring pediatric brain tissue, pediatric brain tumors showed similar NKG2D ligand expression, an activator of NK cells, and NK cell infiltration. However, when compared to adult brain tumors, pediatric brain tumors showed a less immunosuppressive tumor microenvironment.
Immunological profiling of mutational and transcriptional subgroups in pediatric and adult high-grade gliomas	Bockmayr et al.	Pediatric brain development may elicit a different immune profile than in adults	There is no associated specific immune cell pattern among pediatric HGGs. Different subtypes within pediatric hGGs exhibited different effects of PD-L1 and CTLA4 expression on survival. In K27-mutated tumors, PD-L1 and CTLA4 correlated with a worse prognosis, while G34-mutated tumors did not see this effect. Additionally, G34-mutated tumors were uniquely found to rely on TGFB1 and HAVCR2 as pathways for immune escape.
Elevated NLR may be a feature of pediatric brain cancer patients	Yalon et al.	Pediatric brain development may elicit a different immune profile than in adults	The neutrophil-to-lymphocyte ratio is significantly elevated in pediatric brain cancer patients.
Pre-treatment lymphopenia and indication of tumor-induced systemic immunosuppression in medulloblastoma	Patel et al.	Pediatric brain development may elicit a different immune profile than in adults	Tumor-induced systemic immune suppression is observed in Mb patients.
Immune gene and cell enrichment is associated with a good prognosis in ependymoma	Donson et al.	The BBB makes pediatric brain tumors immunologically "cold"	Increased BBB- and tumor-infiltrating immune cells, such as CD4+ T cells, were associated with improved survival and a status of non-recurrence in ependymomas.
Classification of pediatric gliomas based on immunological profiling: implications for immunotherapy strategies	Wang et al.	The BBB makes pediatric brain tumors immunologically "cold"	Pediatric gliomas that were immunologically "cool" had a poor prognosis and responded poorly to immunotherapy because of inadequate immune infiltration.
Integrated proteogenomic characterization across major	Petralia et al.	The BBB makes pediatric brain tumors immunologically "cold"	5 distinct proteomic immune signatures were elicited from broad pediatric brain cancer profiling. Several genes, including GRIA1, CAMK2A/B/G/D, PIK3CA, and ADAP1, were highly elevated in HGG,

histological types of pediatric brain cancer			LGG, and gangliomas. This subset has been shown to actively participate in glutamate signaling, a transduction pathway that has been linked to the abnormal expression of adenosine makers such as phosphatases ENTPD1 and NT5E in a number of malignancies.
Characterization of distinct immunophenotypes across pediatric brain tumor types	Griesinger et al.	The BBB makes pediatric brain tumors immunologically “cold”	When compared to GBM, Mb, or normal tissue, PA and EPN tumors showed both a much higher population of infiltrating immune cells and a population of activated myeloid cell-skewed functional phenotype. In contrast, normal tissue-like GBM and Mb showed fewer infiltrating leukocytes and more subdued myeloid characteristics.
Subtype and grade-dependent spatial heterogeneity of T-cell infiltration in pediatric glioma	Robinson et al.	The BBB makes pediatric brain tumors immunologically “cold”	When compared to HGG, low-grade tumors showed larger T cell counts, although T cell infiltration varied by subtype, with PXA and ganglioglioma having comparatively higher T cell densities.
Exploring DNA Methylation for Prognosis and Analyzing the Tumor Microenvironment in Pleomorphic Xanthoastrocytoma	Tang et al.	The BBB makes pediatric brain tumors immunologically “cold”	In comparison to gangliomas, PXAs displayed considerably greater CD8+ T cell signatures.
Pilocytic astrocytoma demethylation and transcriptional landscapes link bZIP transcription factors to immune response	Aichmuller et al.	The BBB makes pediatric brain tumors immunologically “cold”	There is a significant immune infiltration variation among PA tumors.
Preoperative systemic levels of VEGFA, IL-7, IL-17A, and TNF-β delineate two distinct groups of children with brain tumors	Sandén et al	Pediatric brain development requires immunosuppression	Children with brain tumors' systemic immune profiles were examined, and it was revealed that although Mb patients had a distinct cytokine profile, most tumor diagnoses were unable to consistently elicit a specific systemic immunological status.
Tumor mutational burden and driver mutations: Characterizing the genomic landscape of pediatric brain tumors	Patel et al.	Lower mutational burden in pediatric brain tumors	TMB was low in 91.8 percent of the 723 pediatric brain tumors studied by complete genetic profiling.
Tumor mutation burden, DNA mismatch repair status and checkpoint immunotherapy markers in primary and relapsed malignant rhabdoid tumors	Abro et al.	Lower mutational burden in pediatric brain tumors, T cell Exhaustion	A TMB of 0.7-1.07/Mb was present in 4 of the 5 primary untreated malignant rhabdoid tumors (MRT), whereas a TMB of 33.81/Mb was present in 1 tumor. The median density of PD-L1-negative CD68+ myeloid cells was highest in ATRT-SHH tumors among the five subgroups, indicating a striking variance in PD1-expressing lymphocyte density among MRTs.
Comprehensive genomic profiling of 282 pediatric low-and high-grade gliomas reveals genomic drivers, tumor mutational burden, and hypermutation signatures	Johnson et al.	Lower mutational burden in pediatric brain tumors	Pediatric LGGs and HGGs both had relatively low TMBs, but HGGs had a hypermutated subpopulation that made up 6% of all HGGs and with more than 20 mutations per Mb.
Tumor antigen precursor protein profiles of adult and pediatric brain tumors identify potential targets for immunotherapy	Zhang et al	Lower mutational burden in pediatric brain tumors	TAPP mRNAs were expressed in 94 percent of adult gliomas vs 55–74 percent of childhood gliomas.
Notch signaling and natural killer cell infiltration in tumor tissues underlie medulloblastoma prognosis	Liang et al.	Lower mutational burden in pediatric brain tumors	Both the prognosis and mutational burden of pediatric Mbs were positively linked with NK infiltration.
Low mutational load in pediatric medulloblastoma still translates into neoantigens as targets for specific T-cell immunotherapy	Blaeschke et al	Lower mutational burden in pediatric brain tumors	Immunogenic antigens were identified in pediatric Mb.

SOX2 immunity and tissue resident memory in children and young adults with glioma	Vasquez et al.	Lower mutational burden in pediatric brain tumors	Immunogenic antigens were identified in pediatric glioma.
High expression of Toll-like receptor 7 is a survival factor in pediatric medulloblastoma	Alvarex-Arellano et al	Recognition	Uric acid, heat shock proteins, ligand transfer molecules generated by CpG DNA, and ECM derivatives acting as toll-like receptor ligands are all danger signals in pediatric brain cancers. TLR7 expression was found to be a prognostic factor in Mb patient survival.
Increased microglia/macrophage gene expression in a subset of adult and pediatric astrocytomas	Engler et al.	Recognition	Microglia/macrophage-related genes are selectively enriched in pediatric GBM tumors of the mesenchymal subtype.
Immune cell infiltration and cytokine secretion analysis reveal a non-inflammatory microenvironment of medulloblastoma	Diao et al.	Leukocyte Recruitment & Infiltration	A notably low level of tumor cell infiltration was seen in Mb tumors.
Immunocytochemical detection of leukocyte-associated and apoptosis-related antigen expression in childhood brain tumors	Bodey et al.	Leukocyte Recruitment & Infiltration	The expression of CD8+ T cells was shown to be positive in 76.32 percent of Mb, PNET, and astrocytomas, with these cells typically accounting for 1–10 percent of total cells. CD4+ T cells were present in 85.53 percent of brain tumors, making up 1 to 10 percent of all cells found. Finally, macrophages were found in 97.37 percent of brain tumors, accounting for 1–10% of all cells analyzed.
Decreased natural killer cells in diffuse intrinsic pontine glioma patients	Zhang et al.	Leukocyte Recruitment & Infiltration	Patients with DIPG have considerably lower NK cell levels and higher B cell levels compared to control blood samples.
Know your neighbors: Different tumor microenvironments have implications in immunotherapeutic targeting strategies across MB subgroups	Pham et al.	Leukocyte Recruitment & Infiltration, Monocyte Polarization	There exists variation between subtypes of Mb, with murine SHH-group tumors experiencing the greatest infiltration with immune cells. The proportions of MDSCs and TAMs in murine Mb subgroups were extremely different, with SHH-group tumors exhibiting the highest degree of infiltration.
Prognostic relevance of tumor-infiltrating lymphocytes and immune checkpoints in pediatric medulloblastoma	Vermeulen et al.	Equilibrium	Pediatric Mb tumors exhibit downregulation of MHC-I and CD1. SERPINB1 and SERPINB4 are granzyme inhibitors that were acquired by 23% and 50%, respectively, of Mb tumors.
Pediatric primitive neuroectodermal tumors of the central nervous system differentially express granzyme inhibitors	Vermeulen et al.	Equilibrium	Pediatric primitive neuroectodermal tumors exhibit downregulation of MHC-I and CD1. SERPINB9, SERPINB1, and SERPINB4 were expressions in 29%, 29%, and 57% of CNS-PNET tumors, respectively.
IDO1 involvement in mTOR pathway: a molecular mechanism of resistance to mTOR targeting in medulloblastoma	Folgiero et al.	Immunosuppressive Signalling Cascades	Mutations in medulloblastoma cells particularly increased IDO1 expression by mTOR. IDO1 plays a significant role in the development of Tregs.
T-cell TGF-β signaling abrogation restricts medulloblastoma progression	Gate et al.	Immunosuppressive Signalling Cascades	The elimination of Treg cells and an enhancement in CD8+ T cells' ability to perform antibody-dependent cell-mediated cytotoxic tasks were both caused by the suppression of TGF- signaling.
Medulloblastoma rendered susceptible to NK-cell attack by TGFβ neutralization	Powell et al.	Immunosuppressive Signalling Cascades	TGF- in Mb is crucial in preventing NK from destroying tumor cells without prior antigen identification
Medulloblastoma exosome proteomics yield functional roles for extracellular vesicles	Epple et al.	Immunosuppressive Signalling Cascades	Low levels of TEX addition led to reduced levels of IFN-y secretion, whereas high levels of TEX addition led to high amounts of IFN-y.

Proteomic and immunologic analyses of brain tumor exosomes	Graner et al.	Immunosuppressive Signalling Cascades	Exosomes from pediatric gliomas also carried heat-shock proteins in addition to the immunosuppressive cytokine TGF-β.
Increased expression of tumor-associated antigens in pediatric and adult ependymomas: Implication for vaccine therapy	Yeung et al.	Immunosuppressive Signalling Cascades	Pediatric EPN frequently express EphA2, IL-13Rα2, and Survivin.
Molecular analyses reveal inflammatory mediators in the solid component and cyst fluid of human adamantinomatous craniopharyngioma	Donson et al.	Immunosuppressive Signalling Cascades	There was a particularly enriched pro-inflammatory cytokine pattern in fluid from pediatric adamantinomatous craniopharyngioma compared to fluids from pilocytic astrocytoma.
B7–H3 as a Prognostic Biomarker and Therapeutic Target in Pediatric central nervous system Tumors	Maachani et al.	Immunosuppressive Signalling Cascades	B7-H3 is widely expressed in a variety of pediatric CNS tumors, with its aberrant expression correlating with tumor grade. High B7-H3 mRNA expression is significantly correlated with poorer survival in various brain tumor types of childhood.
Aberrant immunostaining pattern of the CD24 glycoprotein in clinical samples and experimental models of pediatric medulloblastomas	Sanden et al.	Monocyte Polarization	Pro-tumor M2-polarized TAMs in pediatric brain tumors can be detected through CD24 specifically.
NF-κB upregulation through epigenetic silencing of LDOC1 drives tumor biology and specific immunophenotype in Group A ependymoma	Griesinger et al.	Monocyte Polarization	The NF-κB pathway is aberrantly increased in Group A EPN.
Platelet-derived growth factor beta is a potent inflammatory driver in paediatric high-grade glioma	Ross et al.	Monocyte Polarization	Shorter rodent median survival and higher cytokine production in pHGG were accompanied with an increase in TAM infiltration of PDGFB-driven tumors.
Clinical features and prognosis of pediatric infradiaphragmatic craniopharyngioma relative to the tumor inflammatory response	Peng et al.	Monocyte Polarization	Infradiaphragmatic craniopharyngiomas demonstrate higher levels of the cytokines IL-8 and IL-6
Therapeutic implications of CD1d expression and tumor-infiltrating macrophages in pediatric medulloblastomas	Teo et al.	Monocyte Polarization	Apart from CD1d, Mb-infiltrating macrophages express CD163.
Tumour-associated macrophages exhibit anti-tumoural properties in Sonic Hedgehog medulloblastoma	Maximov et al.	Monocyte Polarization	In SHH Mb, TAMs exhibit anti-tumoral capabilities.
Immunocytochemical detection of leukocyte-associated and apoptosis-related antigen expression in childhood brain tumors	Bodey et al.	Escape from Apoptosis	The death receptor is expressed by the vast majority of pediatric glial malignancies, especially astrocytomas.
PD-1/PD-L1 and immune-related gene expression pattern in pediatric malignant brain	Hwang et al.	T cell Exhaustion	40 percent of AT/RT, 20 percent EPN, and 19 percent HGG patients had PD-L1 expression, but PD-L1 expression was not observed in

tumors: clinical correlation with survival data in Korean population			Mb. There was no relationship between PD-L1 and survival in AT/RT, EPN, high grade gliomas, and Mb patients.
PD-L1 expression in pediatric low-grade gliomas is independent of BRAF V600E mutational status	Martin et al.	T cell Exhaustion	Low PD-1L expression (5%) was seen in the majority of low-grade gliomas, and PD-1L expression was not dependent on BRAF V600E mutations.
PD-L1 expression in medulloblastoma: an evaluation by subgroup	Martin et al.	T cell Exhaustion	PD-L1 expression was present in only some Mb subtypes; in particular, the SHH subtype exhibited substantial PD-L1 expression.
The role of clinical factors and immunocheckpoint molecules in the prognosis of patients with supratentorial extraventricular ependymoma: a single-center retrospective study	Wang et al.	T cell Exhaustion	Positive for PD-L1 was linked to a worse progression-free survival rate. Additionally, at 3, 5, and 10 years, patients with PD-L1 positivity had recurrence hazard ratios that were, respectively, 10.445, 5.539, and 3.949 times higher than those of patients with PD-L1 negativity.
Specific expression of PD-L1 in RELA-fusion supratentorial ependymoma: Implications for PD-1-targeted therapy	Witt et al.	T cell Exhaustion	Particularly high PD-L1 levels were present on both the tumor cells and myeloid cells in supratentorial RELA fusion (ST-RELA) tumors.
Identification and analyses of extra-cranial and cranial rhabdoid tumor molecular subgroups reveal tumors with cytotoxic T cell infiltration	Chun et al.	T cell Exhaustion	MRTs displayed greater numbers of PD-L1-expressing CD68+ myeloid cells as well as higher PD-L1 expression than ATRTs.
PD-1/PD-L1 expression in a series of intracranial germinoma and its association with Foxp3+ and CD8+ infiltrating lymphocytes	Liu et al.	T cell Exhaustion	PD-1 was expressed in 96% of tumor-infiltrating lymphocytes of intracranial germinomas, whereas PD-L1 was expressed in 92% of tumor cells.
